# Short- and long-term effects of ^56^Fe irradiation on cognition and hippocampal DNA methylation and gene expression

**DOI:** 10.1186/s12864-016-3110-7

**Published:** 2016-10-24

**Authors:** Soren Impey, Timothy Jopson, Carl Pelz, Amanuel Tafessu, Fatema Fareh, Damian Zuloaga, Tessa Marzulla, Lara-Kirstie Riparip, Blair Stewart, Susanna Rosi, Mitchell S. Turker, Jacob Raber

**Affiliations:** 1 Oregon Stem Cell Center and Department of Pediatrics, Oregon Health and Science University, Portland, OR 97239 USA; 2 Department of Cell, Developmental Biology, and Cancer Biology, Oregon Health and Science University, Portland, OR 97239 USA; 3 Brain and Spinal Injury Center, University of California, San Francisco, San Francisco, CA 94110 USA; 4 Departments of Physical Therapy Rehabilitation Science, University of California, San Francisco, San Francisco, CA 94110 USA; 5 Neurological Surgery, University of California San Francisco, Zuckerberg San Francisco General Hospital, San Francisco, CA 94110 USA; 6 Department of Behavioral Neuroscience, Oregon Health and Science University, Portland, OR 97239 USA; 7 Oregon Institute of Occupational Health Sciences and Department of Molecular and Medical Genetics, Oregon Health and Science University, Portland, OR 97239 USA; 8 Departments of Neurology and Radiation Medicine, Oregon Health and Science University, Portland, OR 97239 USA; 9 Division of Neuroscience ONPRC, Oregon Health and Science University, Portland, OR 97239 USA

**Keywords:** Hippocampus, DNA methylation, Cognition, Network stability, Gene expression

## Abstract

**Background:**

Astronauts are exposed to ^56^Fe ions that may pose a significant health hazard during and following prolonged missions in deep space. We showed previously that object recognition requiring the hippocampus, a structure critical for cognitive function, is affected in 2-month-old mice irradiated with ^56^Fe ions. Here we examined object recognition in 6-month-old mice irradiated with ^56^Fe ions, a biological age more relevant to the typical ages of astronauts. Moreover, because the mechanisms mediating the detrimental effects of ^56^Fe ions on hippocampal function are unclear, we examined changes in hippocampal networks involved in synaptic plasticity and memory, gene expression, and epigenetic changes in cytosine methylation (5mC) and hydroxymethylation (5hmC) that could accompany changes in gene expression. We assessed the effects of whole body ^56^Fe ion irradiation at early (2 weeks) and late (20 weeks) time points on hippocampus-dependent memory and hippocampal network stability, and whether these effects are associated with epigenetic changes in hippocampal DNA methylation (both 5mC and 5hmC) and gene expression.

**Results:**

At the two-week time point, object recognition and network stability were impaired following irradiation at the 0.1 and 0.4 Gy dose, but not following irradiation at the 0.2 Gy dose. No impairments in object recognition or network stability were seen at the 20-week time point at any irradiation dose used. Consistent with this pattern, the significance of pathways for gene categories for 5hmC was lower, though not eliminated, at the 20-week time point compared to the 2-week time point. Similarly, significant changes were observed for 5mC gene pathways at the 2-week time point, but no significant gene categories were observed at the 20-week time point. Only the 5hmC changes tracked with gene expression changes.

**Conclusions:**

Dose- and time-dependent epigenomic remodeling in the hippocampus following ^56^Fe ion exposure correlates with behavioral changes.

**Electronic supplementary material:**

The online version of this article (doi:10.1186/s12864-016-3110-7) contains supplementary material, which is available to authorized users.

## Background

A unique feature of the space radiation environment is the presence of galactic cosmic rays (GCR) and solar particle events (SPE). The former involves protons and fully ionized atomic nuclei such as ^56^Fe, while the latter includes predominantly low to medium energy protons with a small heavy ion component. These radiation exposures may pose a significant hazard to deep space flight crews during and following the mission. The hazards associated with the space environment will likely impact many organ systems. In the central nervous system (CNS), radiation exposure significantly affects the hippocampus [1–18], a structure critical for memory function. For example, object recognition memory [19], which uses a 24 h interval between learning and memory assessment to test hippocampal function [20], is sensitive to effects of irradiation [21]. Object recognition memory is impaired in 2-month-old mice two weeks following ^56^Fe ion irradiation [22] and twelve weeks following irradiation with protons [23]. It is unclear whether object recognition is also sensitive to effects of charged particles in mice irradiated at 6 months of age, a biological age relevant to the typical ages of astronauts during space missions.

The mechanisms mediating the detrimental effects of a charged particle exposure such as ^56^Fe ions on hippocampus-dependent cognitive function are not clear, but might be associated with changes in hippocampal networks involved in synaptic plasticity and memory. Age-related changes in immediate early gene Activity-Regulated Cytoskeleton-Associated Protein (*Arc*) in the hippocampus have been reported [24]. Our work showed that head-only ^56^Fe ion irradiation of two-month-old mice alters hippocampal expression of *Arc* [25, 26]. *Arc* expression has been used to provide important insight into the post-transcriptional infrastructure of gene expression involved in synaptic plasticity and memory [27] (for review, [28]). When neurons are engaged in information processing, *Arc* is rapidly transcribed and can be visualized and quantified after ~5 min. Subsequently, the mRNA is translocated to the cytoplasm where it remains detectable for ~20–30 min after the initial transcription. Thus, two different cellular compartments (nuclear and cytoplasmic) can be clearly distinguished, providing means to identify which neurons were active during distinct behavioral experiences [27].

*Arc* mRNA localization is a useful tool to test memory function. During spatial exploration, when an animal enters a specific spatial location to which the cell responds (known as the cell's place field), hippocampal pyramidal neurons in the CA areas display robust firing. The place fields create an environment-specific "map", which is believed critical in establishing the context of a memory [29, 30]. Place fields are highly reproducible and stable across multiple visits to the same environment. However, this stability is not seen when different environments are visited. Analysis of the temporal kinetics of *Arc* mRNA allows study of neuronal activity and network stability by detecting the number of active cells forming environment-specific maps and capturing the environmental specificity of place fields [27, 31, 32]. When animals visit the same environment twice, ~90 % of cells expressing *Arc* mRNA in the nucleus also express *Arc* mRNA in the cytoplasm, which indicates that the same neurons are active during both explorations. In contrast, when two different environments are visited, the proportion of cells showing double labeling is very low [31, 33].

In addition to network stability, other molecular mechanisms are likely involved in mediating cognitive functions. One of these is DNA methylation because it correlates closely with gene expression. Hippocampal changes in cytosine methylation, a major epigenetic modification involving the addition of a methyl group to cytosine (5mC), play a key role in regulating expression of genes required for spatial learning and memory [34, 35]. A second form of DNA methylation is hydroxymethylcytosine (5hmC), which is derived from 5mC by the action of three Ten-eleven translocation (TET) enzymes (TET 1–3) [36]. TET2 is believed most important for brain function because its levels are higher than those of TET1 or TET3 in this tissue [37, 38]. The levels and localizations of 5mC and 5hmC are high and exceptionally dynamic in the brain during development and aging [39, 40]. The exact roles for 5mC and 5hmC in the brain remain to be elucidated, but their high and dynamic levels in neurons and correlation with gene expression suggest strongly that they play important roles throughout the lifespan.

We recently reported that a single exposure to proton irradiation caused long-term tissue specific changes in DNA methylation patterns that were present 22 weeks following exposure and that mapped to tissue specific pathways (hippocampus versus left ventricle) [41]. That study demonstrated that the brain epigenome is responsive to ionizing radiation. In the current study, we assessed the effects of whole body ^56^Fe ion irradiation at early (2 weeks) and late (20 weeks) time points on hippocampus-dependent memory and hippocampal network stability; and whether these effects are associated with changes in hippocampal DNA methylation (both 5mC and 5hmC) and gene expression.

## Methods

### Animals and study design

Six-month-old C57BL/6 J male mice (*n* = 168 mice in total) were obtained from Jackson Laboratories, Bar Harbor Maine. The biological age of the mice was selected to be relevant to the biological age of astronauts during space missions. The mice were shipped from Jackson Laboratories to Brookhaven National Laboratory (BNL), Upton, New York, and allowed to accommodate to the housing facility there for one week. Subsequently, the mice were irradiated with 0.1, 0.2, or 0.4 Gy of 600 MeV ^56^Fe or sham-irradiated (*n* = 42 mice/radiation dose). For irradiation, mice were individually loaded into 8 × 3 × 3 cm plastic square enclosures with air holes and placed in a foam fixture in the beam line of the NASA Space Radiation Laboratory (NSRL). They were exposed to a rectangular beam of approximately 20 x 20 cm. The focused beam of high-energy was generated by the Booster accelerator at BNL and transferred to the experimental beam line at the NSRL facility. Dose calibration was performed so that the desired dose could be delivered. The exposure time for the 0.1 Gy dose was 0.4 min, the exposure time for the 0.2 Gy dose was 0.7 min, and the exposure time for the 0.4 Gy was 1.1 min. Sham-irradiated mice were placed into the plastic enclosures for the same time as the irradiated mice. Mice were randomly assigned to the experimental groups. The week after the irradiation or sham-irradiation, the mice were shipped to Oregon Health & Science University (OHSU) and were assigned to one of two time points (2 or 20 weeks; *n* = 84 mice/time point). Cognitive testing started two or twenty weeks following irradiation. Following cognitive testing, the mice were killed by cervical dislocation followed by decapitation. The hippocampus of one hemibrain was dissected for DNA methylation and RNA-Seq analyses. The other hemibrain was processed for *Arc* mRNA and TET2 immunohistochemical analyses. All protocols were reviewed and approved by the Institutional Animal Care and Use Committees (IACUC) of OHSU and BNL and were in compliance with all Federal regulations.

### Novel object recognition

The novel object recognition test was performed as described [42]. The mice were habituated to an open field (16 x 16 in., Kinder Scientific, Poway, CA) for 3 times for 10 min each over three subsequent days. On day 4, the mice were placed in the open field containing two identical objects and they were allowed to freely explore for 15 min. On day 5, the mice were placed again in the open field, but one familiar object was replaced with a novel object. The mice were allowed to explore for 15 min. Movement and time spent exploring each object was recorded using Ethovision XT video tracking system (Noldus Information Technology, Sterling, VA) and hand scored by a researcher blinded to the treatment of the mice. The percent time exploring the novel object, out of the total time exploring the novel and familiar objects on day 5, was used to assess novel object recognition. For each group, the preference for the novel versus the familiar object was assessed. The open field arena and objects were cleaned with 5 % acetic acid between mice and trials.

### Water maze

Spatial learning and memory were assessed in the water maze as described [42]. A circular pool (diameter 140 cm) was filled with water made opaque with nontoxic chalk (24 *°*C) and mice were trained to locate a submerged platform. To determine if irradiation affected the ability to swim or learn the water maze task, mice were first trained to locate a clearly marked platform (visible platform, Days 1 and 2). The mice were subsequently trained to locate the platform when it was hidden beneath the surface of opaque water (Days 3–5). Training during the hidden platform sessions (acquisition) required the mice to learn the location of the hidden platform based on extra-maze cues. For both visible and hidden sessions, there were two daily sessions, morning and afternoon, which were 2-h apart. Each session consisted of two trials (with 5-min inter-trial intervals). A trial ended when the mice located the platform. Mice that failed to locate the platform within 60 s were led to the platform by placing a finger in front of their swim path. The mice were taken out of the pool after they were physically on the platform for a minimum of 3 s. During visible platform training, the platform was moved to a different quadrant of the pool for each session. For the hidden platform training, the platform location was kept constant. The mice were placed into the water facing the edge of the pool in one of nine randomized locations. The start location was changed for each trial. The swimming patterns of the mice were recorded with Noldus Ethovision video tracking software (Ethovision XT, Noldus Information Technology, Wageningen, Netherlands) set at six samples/s. The time to locate the platform (latency) and cumulative distance to the target were used as measures of performance for the visible and hidden sessions. Because swim speeds can influence the time it takes to reach the platform, they were also analyzed.

To measure spatial memory retention, probe trials (platform removed) were conducted 1 h after the last hidden trial of each mouse on the first and second day of hidden platform training and 72 h following the last hidden trial of each mouse on the third day of hidden platform training and the cumulative distance to the target location during the probe trials was analyzed.

### Hippocampal network stability

Three days after the last water maze test day, exploration of identical or different environments was used to study the stability of hippocampal networks [27, 31]. The method called catFISH (cellular compartment analysis of temporal activity using fluorescence in situ hybridization) relies on the precise temporal kinetics of the IEG *Arc*, which has been used to provide important insight into the post-transcriptional infrastructure of gene expression involved in synaptic plasticity and memory [27, 31], (for review, [28]). When neurons are engaged in information processing, *Arc* is rapidly transcribed and can be visualized and quantified after ~5 min. Subsequently, the mRNA is translocated to the cytoplasm where it remains detectable for ~20-30 min after the initial transcription. Ultimately, the mRNA is translocated to tagged synapses for protein synthesis. Thus, two different cellular compartments (nuclear and cytoplasmic) can be clearly distinguished, providing means to identify which neurons were active during distinct behavioral experiences [31].

Eight mice from each experimental radiation condition were placed individually into a novel environment (A) and allowed to explore for five minutes, as described [31]. Environment A is a square open field (61 × 61 cm box with 20-cm high walls). After exploration, mice were returned to their cage for 25 min, returned to the same environment for an additional 5 min (AA Paradigm). Another 8 mice of each experimental radiation condition explored environment A for 5 min, and 25 min later they were placed in a different environment (B), a circular arena 45 cm in diameter, and allowed to explore for 5 min (AB Paradigm). Following the last environmental exposure, the mice were killed by cervical dislocation and the brains quickly removed, as described above.

Using catFISH, Arc mRNA appear as discrete nuclear foci (recent transcription ~5-10 min), and/or as diffuse mRNA in the cytoplasm (earlier transcription). Nuclear and cytoplasmic *Arc* can be distinguished using intronic and full-length probe respectively labeled with digoxigenin or fluorescein. *Arc* staining was classified as: a) None (no *Arc* staining); b) cytoplasmic *Arc* staining only; c) nuclear *Arc* staining only; or d) both *Arc*-foci/*Arc*-cyto (containing both foci and cytoplasmic staining). We can determine if the neurons responding (i.e. *Arc*+) to an initial experience (exploration of environment A) are the same neurons that respond to a second and identical experience as expected based on sham-irradiated animals (AA Paradigm), and if the hippocampal networks activated during exploration of the first environment are statistically independent from those activated by the exploration of a different environment (AB Paradigm). For the CA1 and CA3 regions of the hippocampus, the percentage of neurons showing both foci and cytoplasmic staining was analyzed. As the pattern of percentages of *Arc*-positive neurons in both environments might reflect differences in the total number of *Arc*-positive neurons (total number of neurons with *Arc* nuclear foci, cytosolic *Arc*, or both) this number was also determined. For the dentate gyrus, the percent of neurons expressing *Arc* only in the cytoplasm or expressing both *Arc*-foci/*Arc*-cyto (containing both foci and cytoplasmic staining) was analyzed. This measure was analyzed as it reflects the total response to the environment(s).

### Tet2 Immunohistochemistry

Following cervical dislocation and decapitation, hemibrains were rapidly removed and frozen using isopentane and dry ice, as described [26] and stored at −80 °C. The hemibrains were shipped to the University of California, San Francisco, and cut at 20 μm using a cryostat. Slides were sent to OHSU for analysis of Tet2 immunoreacttivity using a specific primary antibody from Santa Cruz Biotechnology (Tet2 S-13, catalog number sc-136926). Briefly, sections (*n* = 3 sections per hemibrain and approximately 200 μm apart) were rinsed in phosphate buffered saline (PBS), and incubated in 4 % normal goat serum (NGS) in PBS with 0.4 % triton X-100 (PBS-TX). Next, sections were incubated overnight with primary antibodies against Tet2 (1:250) in 4 % NGS in PBS-TX. The next day, tissue sections were washed in PBS, incubated for 2.5 h in donkey anti-rabbit Alexa 488 (1:200) in 4 % NGS in PBS-TX and again rinsed in PBS. Analysis of Tet2 immunoreactivity was performed using an Olympus IX81 confocal microscope equipped with Slidebook software. Images of hippocampal regions (CA1, CA3, and dentate gyrus) and the cortex were captured within 3 sections (Bregma −1.58 to −2.46) using a 20x objective (UPlan FL, Olympus). Tet2 immunoreactivity was quantified within fixed area frames; CA1 (box, 125 × 50 μm), CA3 (2 boxes, 95 × 85 μ m each), dentate gyrus (2 boxes, 95 × 85 μm each), and cortex (posterior parietal association area; box, 240 × 200 μm). Background threshold levels were set and applied to all images for comparison. Pixel intensities above this threshold were used for quantification measures (area occupied by pixels and intensities of pixels). The total intensity was also quantified as a measure of overall pixel intensity within a specific brain region.

### DNA methylation

DNA was isolated from the hippocampus. Antibodies against 5mC and 5hmC were used to immunoprecipitate sonicated DNA preparations for methyl-DNA immunoprecipitation (meDIP) and hydroxymethyl-DNA immunoprecipitation (hmeDIP, respectively, from twelve pools of tissues (6 x 2 pools of hippocampal tissues or 2 pools/radiation condition/time point). These antibodies were used to precipitate genomic regions that are enriched for either 5mC or 5hmC. Following immunoprecipitation, high-throughput genomic sequencing and segmentation analyses were used to identify enriched genomic regions. For DIP-Seq library preparation, RNAse-treated DNA was isolated using the Qiagen Allprep DNA/RNA protocol. The DNA was sonicated using a Cole Parmer CPX-132 sonicator (75 % amplitude, 3×10’) and polished using the DNA terminator end repair kit (Lucigen). DNA fragments were A-tailed using Klenow exo- (Epicenter) and ligated to un-methylated HT TrueSeq indexed adapters and purified. The resulting purified DNA was denatured at 95 °C, resuspended in 100 ul of DIP IP buffer, and immunoprecipitated with 1 μg of the highly specific 5-methylcytosine antibody (EMD Millipore) or 2 ul of 5-hydroxymethylcytosine (Active Motif) antibody and Dynal anti-mouse IgG beads. Beads were rinsed 7 times with IP buffer, eluted with 1 % SDS at room temperature and the eluted DNA purified and subjected to limited amplification (~18 cycles). Libraries were sequenced on the HiSeq2000 platform at the OHSU Massively Parallel Sequencing Shared Resource or the Oregon State University Center for Genome ReseArch. DIP-Seq regions methylated above “background” were identified using an optimized sliding window parameter and enriched regions selected over background models via a Montecarlo-permutation test [43].

### RNA-Seq

To facilitate direct comparison of DIP-Seq data with gene expression data, RNA-Seq was used to profile transcription from the same animals used for the DIP-Seq experiments. RNA was isolated using the NEBnext poly A selection kit (New England Biolabs). Illumina high-throughput sequencing technology was used to profile RNA levels in an unbiased manner. Differential methylation may occur at novel regions and the unbiased nature of RNA-Seq analyses enables analysis of associated un-annotated transcription. For Illumina RNA-Seq library preparation, we used the NEBnext Ultra kit according to the manufacturers specifications (New England Biolabs).

Illumina data were mapped to the UC Santa Cruz assembly using Bowtie [44]. For RNA-Seq analyses, tags that overlap with known RefSeq (UCSC RefSeq annotation) were counted using R scripts [45]. Significance was assessed using the DESeq2 package [46]. The Storey Q-test was used to adjust for multiple comparisons [47].

Libraries were sequenced on the HiSeq2000 platform at the OHSU Massively Parallel Sequencing Shared Resource.

### Real time PCR

For real-time PCR, 20–22 bp primers were designed using MIT's Primer3 software (http://bioinfo.ut.ee/primer3/) using standard parameters and all quantitation utilized standard curve real-time PCR. Primers will be provided upon request. PCRs (10 μl) contained 1 μl 10× PCR buffer (Invitrogen), 2.5 mM MgCl_2_, 200 μM dNTP (Roche), 0.125–0.25 μM primer (IDT), 1× SYBR green I (Invitrogen), and 1 U platinum *Taq* (Invitrogen). PCR was run on an Opticon OP346 (MJ Research) for one cycle at 95 °C, 35 s, and 30–50 cycles at 94 °C, 15 s; 68 °C–70 °C, 40 s. RT-PCR experiments were normalized to 18S RNA levels (other housekeeping genes showed similar results) and were expressed as ng of gel-purified (Qiagen) amplicon.

### Bioinformatics and statistical analyses

All behavioral, cognitive, and *Arc* data are shown as mean ± SEM. The statistical analyses of the data were performed using SPSS™ (Chicago, IL) and GraphPad Prism™ (San Diego, CA) software packages. To compare effects of radiation across groups, ANOVA was used with irradiation as the between factor, followed up by Dunnett’s posthoc tests or *t*-tests when appropriate. To analyze locomotor activity over three days, repeated measures ANOVA was used. To assess effects of radiation on *Arc* measures across dose conditions in mice exposed to a specific environmental condition, one-way ANOVAs were used. To compare exploration of the objects and the percentage of *Arc*-positive cells and total number of *Arc* cells following exposure to the two different environmental conditions, 2-sided *t*-tests were used. All figures were generated using GraphPad Prism software. We considered *p* < 0.05 as statistically significant.

Single read sequence data was mapped to the mouse reference genome (UCSC mm9) using the Bowtie algorithm using standard flags except for allowing 2 mismatches [44]. Sequences that map to a single location were selected and domains enriched for 5mC or 5hmC were selected using a parameter-optimized Monte-Carlo-based segmentation algorithm [43]. A 1000 bp sliding-window was selected based on iterative analyses that maximized the number of enriched regions. A comparison of different high-throughput sequencing based methods to study DNA methylation concluded that MeDIP-Seq covers ~ 67 % of genomic CpGs [48].

For statistical comparisons of biological samples, regions of methylation enrichment were merged and differences in methylation interrogated with FDR-adjusted chi-square or negative binomial statistics [49]. Statistical and visualization studies involved the R programming language and Bioconductor packages [49]. Gene ontology analyses utilized the Bioconductor Goseq package, which adjusts for RNA-Seq length bias artifacts [50]. For gene ontology analyses the top 2000 DMRs (differentially methylation regions) or DHRs (differentially hydroxymethylated regions) (FDR-adjusted *p* < 0.01) within 50 kb of a transcriptional start site were non-redundantly annotated. Unless otherwise stated, overlap between DMRs and RNA-Seq data was analyzed using a similar relational approach.

Pathway analyses involved standard bioconductor packages (e.g. cmap, keggraph, gsea). DIP sequence-tag heatmaps were generated in R by plotting median-normalized DIP-Seq tag density in gene bodies and indicated flanking regions with color-maps scaled to the 80 % quantile. Statistical analyses of pathway data were conducted via FDR-adjusted Fisher exact or KS-tests. We considered (FDR-adjusted) *p* < 0.01 as statistically significant.

## Results

### Effects of ^56^Fe ion irradiation on cognitive performance

**Fig. 1:**
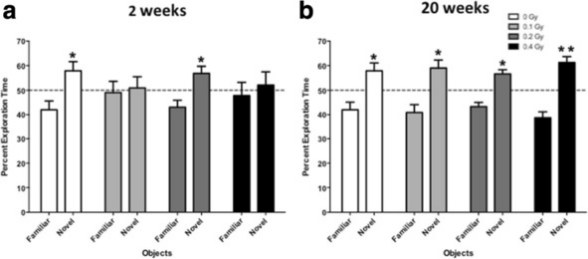
**a** Object recognition at the 2-week time point. Sham-irradiated mice and those irradiated with ^56^Fe ions (600 MeV) at 0.2 Gy showed object recognition and explored the novel object more than the familiar one but mice irradiated with either 0.1 Gy or 0.4 Gy did not. **b** Object recognition at the 20-week time point. All groups explored the novel object significantly more than the familiar one. *N* = 16 mice/dose. *p < 0.05; **p < 0.01 versus familiar object

When activity levels in the open field over three days were analyzed at 2 and 20 weeks following sham-irradiation and irradiation, all groups habituated to the open field and showed higher activity levels in the open field on the first day than on subsequent days and there was a small, but significant effect of ^56^Fe ion irradiation at some doses at the 20 week time point (Additional file 1: Figure S1). When object recognition was assessed at the 2-week time point, detrimental effects of ^56^Fe ion irradiation were seen with a non-linear dose response (Fig. 1a). Mice irradiated with either 0.1 Gy or 0.4 Gy were impaired in object recognition and showed no preference for exploring the novel object. In contrast, sham-irradiated mice and those irradiated with 0.2 Gy showed object recognition and explored the novel object significantly more than the familiar one. Interestingly, the detrimental effects of ^56^Fe ion irradiation on object recognition were transient because all dose groups explored the novel object significantly more than the familiar one at the 20-week time point (Fig. 1b). In contrast to object recognition, no group differences were observed for spatial learning and memory in the water maze at either time point.

### Effects of ^56^Fe ion irradiation on network stability in the CA1 region of the hippocampus

**Fig. 2:**
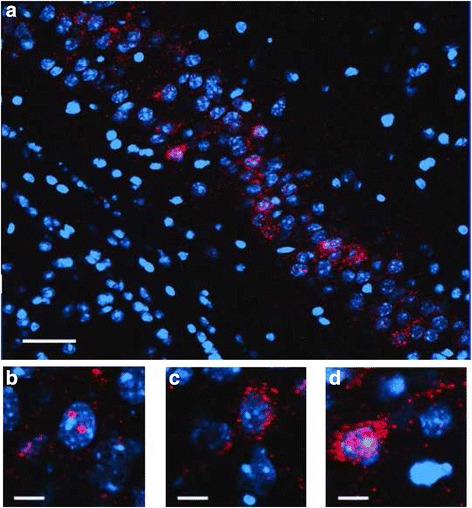
Representative images for Arc catFISH data in area CA3. **a** Fluorescence image showing Arc expression following exploration of identical (AA) environments (image taken at 20× 1 z stack). Scale bar: 50 μm. Example of neurons with foci only (**b**), cytoplasmic only (**c**) or cytoplasmic and foci Arc expression (**d**) (Scale bar: 100 μm). The Arc mRNA is illustrated in red and cell nuclei are indicated in blue (DAPI)

**Fig. 3:**
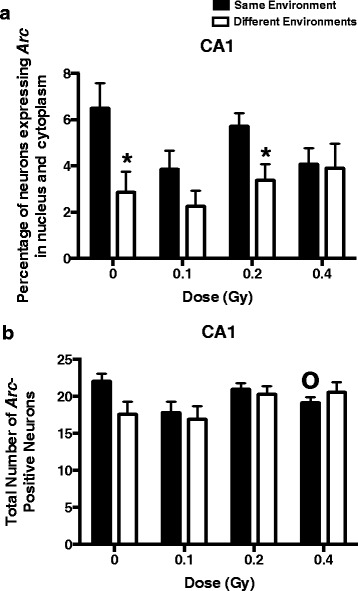
**a** The percentage of *Arc*-positive neurons in the CA1 region of the hippocampus at the 2-week time point. The percentage of *Arc*-positive neurons in the CA1 region of the hippocampus of sham-irradiated mice and mice irradiated with ^56^Fe at 0.2 Gy was higher following exposure to twice the same environment than two different environments. **b**. The total number of *Arc*-positive neurons in the CA1 region of the hippocampus at the 2-week time point. There was no significant difference in the total number of *Arc*-positive neurons following exposure to twice the same environment than two different environments but there was a significant difference between 0 and 0.4 Gy. *p < 0.05 versus same environment; ^0^ *p* < 0.05 versus 0 Gy. *N* = 5-8 mice/dose/environment

To determine whether the impairments in object recognition were associated with reduced ability of hippocampal neurons to recognize similar environments and to discriminate distinct environments, we assessed the effects of ^56^Fe ion irradiation on network stability. This ability was analyzed by assessing the percentage of *Arc*-positive neurons expressing both *Arc*-positive foci in the nucleus and *Arc* staining in the cytoplasm. This combination represents the population of neurons activated by both experiences (see Fig. 2 for representative images). At the 2-week time point, the percentage of *Arc*-positive neurons expressing *Arc* mRNA in the nucleus and cytoplasm in the CA1 region of the hippocampus of sham-irradiated mice and mice irradiated with ^56^Fe at 0.2 Gy was significantly higher following exposure twice to the same environment, as opposed to exposure to two different environments (Fig. 3a). In contrast, the percentage of dual *Arc*-positive neurons in the CA1 region of the hippocampus was not different in these two environmental conditions in mice irradiated with 0.1 (*t* = 1.556, *p* = 0.1433) or 0.4 Gy (Fig. 3a). Thus, the *Arc* mRNA and the novel object recognition results were consistent because in both cases significant radiation effects were observed for the 0.1 and 0.4 Gy dose groups, but not for the 0.2 Gy dose group. There were also trends towards an effect of dose on the percentage of *Arc*-positive cells following exposure twice to the same environments (*p* = 0.0879), a difference between 0 and 0.1 Gy (*p* = 0.0758) and 0 and 0.4 Gy (*p* = 0.0739), but these did not reach significance.

As the pattern of percentages of *Arc*-positive neurons in both environments might reflect differences in the total number of *Arc*-positive neurons (total number of neurons with *Arc* nuclear foci, cytosolic *Arc*, or both), this number was also determined (Fig. 3b). No significant difference in the total number of *Arc*-positive neurons was observed following exposure twice to the same environment, as compared with the two different environments. A trend towards a higher total number of *Arc*-positive neurons was observed in sham-irradiated mice, but it did not reach significance (*p* = 0.0692). However, similar to what was seen for the percentage of *Arc*-positive neurons (Fig. 3a), there were also trends towards an effect of dose on the total number of *Arc*-positive cells following twice exposure to the same environments (*p* = 0.0619), a difference between 0 and 0.4 Gy (*p* = 0.0481) and a trend towards a difference between 0 and 0.1 Gy (*p* = 0.0535). These data indicate that group differences in the total number of *Arc*-positive cells cannot account for the pattern of the percentage of *Arc*-positive neurons in the CA1 region of the hippocampus.

**Fig. 4:**
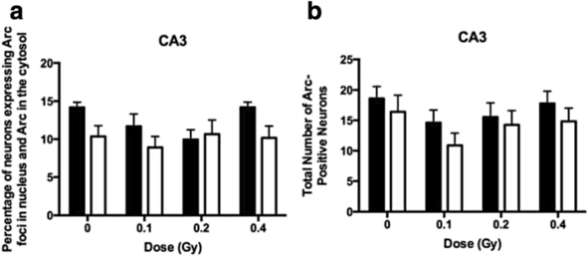
The percentage of Arc-positive neurons in the CA3 region of the hippocampus at the 2-week time point. There were no effects of dose or trend towards group difference between dose conditions in mice following exposure to twice the same environment and following exposure to different environments. **b** The total number of Arc-positive neurons in the CA3 region of the hippocampus at the 2-week time point. There were no effects of dose or trend towards group difference between dose conditions in mice following exposure to twice the same environment and following exposure to different environments. *N* = 5-8 mice/dose/environment

The significant reduction in levels of dual cytoplasmic and nuclear *Arc* staining observed in the CA1 region of sham irradiated mice exposed to different environments, as compared to the same environment twice (Fig. 3a), was not observed in the CA3 region of the hippocampus (Fig. 4).

Expression of Arc protein in the hippocampal dentate gyrus was altered in mice irradiated with ^56^Fe ions at two months of age [25, 26]. To determine whether ^56^Fe ion irradiation also affect *Arc* mRNA levels in the dentate gyrus, we analyzed the total number of *Arc*-positive neurons in the dentate gyrus activated by both behavioral exposures. There was no effect of radiation on the percent of neurons expressing *Arc* only in the cytoplasm or expressing both *Arc*-foci and showing cytoplasmic staining in the dentate gyrus following exposure to twice the same environment or two different environments (Additional file 2: Table S1). These data suggest that the changes reported before may only occur at the translational level in de dentate gyrus.

At 20 weeks following ^56^Fe irradiation, there were no effects of radiation on the percentage of *Arc*-positive neurons or the total number of *Arc*-positive neurons in the CA1 region of the hippocampus or a difference in the percentage of *Arc*-positive neurons following exposure to twice the same environment than two different environments.

### DNA methylation in hippocampus following sham and ^56^Fe irradiation

**Fig. 5:**
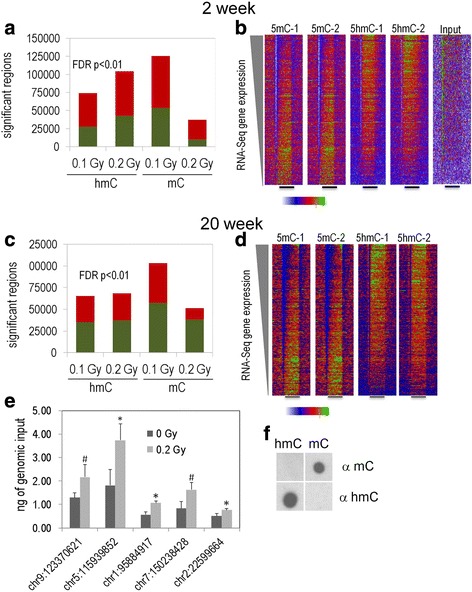
Number of genetic regions with significantly increased (green) and decreased (red) 5hmC and 5 mC at the 2- (**a**) and 20-week (**c**) time points. At both time points, there were more regions with up- and downregulated 5mC at the 0.1 than 0.2 Gy. Dip density histograms illustrating 5mC and 5hmC signal at RefSeq genes sorted by RNA-Seq gene expression levels at the 2-week (**b**) and 20-week (**d**) time points. **e** Real-time PCR validation of significantly upregulated DIP-Seq regions at the 0.2 Gy 2wk time point (samples selected based on ranked *p*-value). Real-time PCR was conducted with genomic input standard curves and 4 DIP biological replicates. *denotes *p* < 0.05 and ^#^denotes *p* < 0.1. **f** The antibodies used to pull down 5mC and 5hmC regions do not cross react

We next assessed whether ^56^Fe ion irradiation is associated with alterations in cytosine methylation (5mC) and/or cytosine hydroxymethylation (5hmC) in the hippocampus. As there were profound differences in the effects of 0.1 Gy and 0.2 Gy ^56^Fe ion irradiation on object recognition and network stability in the hippocampus, we compared DNA methylation in these two radiation conditions with sham-irradiation. Figure 5 illustrates regions with significantly increased (green) and decreased (red) 5hmC and 5 mC at the 2- (Fig. 5a) and 20-week time points (Fig. 5c). At both time points, there were more regions with up- and downregulated 5mC at the 0.1 than the 0.2 Gy dose. We validated our replicate 5hmC DIP-Seq analysis and found that the 5 regions tested showed the expected directional trend and that 3 of 5 showed statistically significant increases (Fig 5e). DIP density histograms illustrating 5mC and 5hmC signal at RefSeq genes sorted by RNA-Seq gene expression levels at the 2- and 20-week time points are shown in Fig. 5b and d, respectively. Data shown are from sham-irradiated mice. Data from irradiated mice showed a similar pattern. As expected, there was no evidence of global changes in 5mC or 5hmC signal near RefSeq genes. The antibodies used to pull down 5mC and 5hmC regions do not cross react (Fig. 5f).

**Fig. 6:**
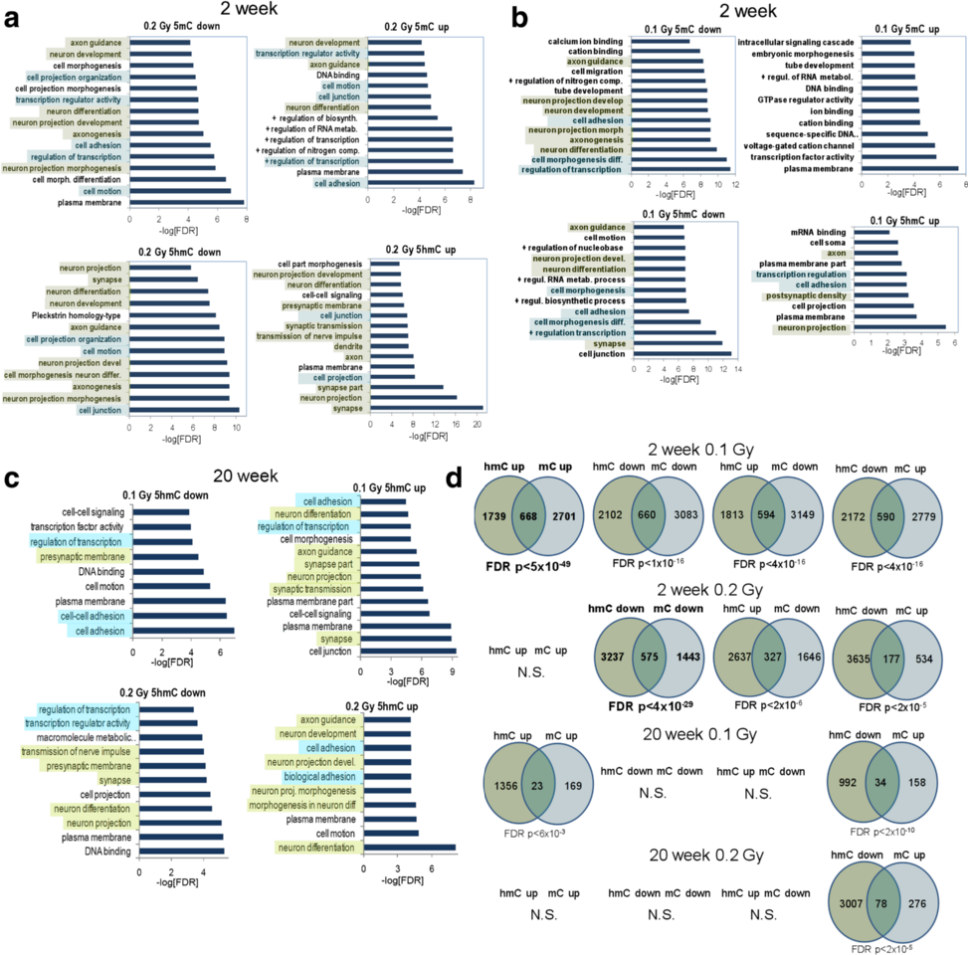
**a** Gene ontology analysis for the 0.1 Gy ^56^Fe dose at the 2-week time point. Regions with decreased 5mC were associated with pathways in neuronal categories linked to axon guidance, axogenesis, neuronal development and differentiation. There was an overlap in non-neuronal categories ^56^Fe ion irradiation with that seen 22 weeks following proton irradiation. Regions with both decreased and increased 5hmC had highly significant overlap with pathways linked to decreased 5mC. Green highlights neuronal categories and blue highlights non-neuronal categories also observed 22 weeks following proton irradiation. **b** Gene ontology analysis for the 0.2 Gy ^56^Fe dose at the 2-week time point. Regions with decreased and increased 5mC were associated with pathways in neuronal categories linked to axon guidance, axogenesis, neuronal development and differentiation. Regions with decreased 5hmC were associated with neuron projection, synapse, neuron differentiation, axon guidance, and axonogenesis. Regions with increased 5hmC were associated with neuron projection development, neuron projection and differentiation, presynaptic membrane, synaptic transmission and synapse, dendrite, axon. **c** Gene ontology analysis at the 20-week time point. Comparing 0.1 and 0.2 Gy, there is an overlap in neuronal categories between regions with upregulated 5hmC (green). Blue highlighted categories are non-neuronal categories that were also observed for the 20-week time point following proton irradiation. The significance of pathways was lower at the 20-week than the 2-week time point. There were no significant gene categories at an FDR < 0.0001 for 5mC. **d** Venn diagrams depict the overlap between significant 5hmC and 5mC DMRs (*q* < 0.01, difference > 50 tags, 5 kb window) at the indicated doses and indicated time points. **a** At the 2-week time point and 0.1 Gy, regions with both decreased and increased 5hmC had highly significant overlap with pathways linked to decreased 5mC. In addition, regions with increase 5hmC had highly significant overlap with pathways linked to increased 5 mC. **b** At the 2-week time point and 0.2 Gy, regions with increased 5hmC had highly significant overlap with pathways linked to decreased 5mC, while regions with decreased 5hmC had highly significant overlap with pathways linked to increased 5mC. **c** At the 20-week time point at 0.1 Gy, regions with decreased 5hmC had highly significant overlap with pathways linked to increased 5mC and regions with increased 5hmC and significant overlap with pathways linked to regions to increased 5mC. **d** At the 20-week time point at 0.2 Gy, regions with decreased 5hmC only had highly significant overlap with pathways linked to increased 5mC

Figure 6a shows the gene ontology analysis for the 0.1 Gy ^56^Fe dose at the 2-week time point. In general, regions with decreased 5mC were associated with pathways in neuronal categories linked to axon guidance, axogenesis, neuronal development and differentiation (Fig. 6a, neuronal categories indicated in green). Blue highlighted categories likely represent a generalized, as opposed to tissue specific, response to radiation exposure [41]. At the 0.1 Gy (Fig. 6a) and 0.2 Gy (Fig. 6b) doses, regions with both decreased and increased 5hmC had highly significant overlap with pathways linked to decreased 5mC. At 0.2 Gy, regions with increased 5hmC had significant overlap with pathways linked to decreased 5mC, while regions with decreased 5hmC had highly significant overlap with pathways linked to increased 5mC (Fig. 6b). Regions with decreased 5mC also showed less enrichment for categories linked to neuronal function or maturation. Interestingly, ontology categories involved in transcriptional regulation, cell adhesion, and cell morphogenesis were more significantly represented at 0.1 Gy, while gene categories linked to neuronal process growth and maturation were overrepresented at 0.2 Gy. In total, the results from the 2-week time point suggest that the dynamic regulation of 5hmC near genes correlates with decreases in 5mC.

The gene ontology analysis for 5hmC at the 20-week time point is shown in Fig. 6c. Comparing 0.1 and 0.2 Gy, overlap is observed in neuronal categories between regions with upregulated 5hmC (green). Blue highlighted categories are non-neuronal categories enriched across conditions that likely represent a generalized response to radiation exposure [41]. In general, the FDR-adjusted significance of shared neuronal and non-neuronal pathways was several orders of magnitude lower at the 20-week than the 2-week time point (Fig. 6c versus Fig. 6a and b). Nonetheless, a striking observation for 5hmC changes, particularly for the 0.2 Gy dose, is that the neuronal gene ontology pathways affected at 2 weeks remain altered at 20 weeks. This observation demonstrates stability over time for 5hmC changes induced by the radiation exposure. In contrast, there were no significant gene categories at an FDR-adjusted *p* <0.001 for 5mC. In total, the DNA methylation data from the two doses and two time points suggest that changes in 5mC (associated with gene pathways) seen at the 2-week time point were mostly transient, while 5hmC changes were mostly stable.

We next examined the relationship between individual genes associated with significant changes in 5mC or 5hmC (Fig. 6d). Genes associated with up or down-regulated 5mC regions largely showed highly significant overlap with up or down-regulated 5hmC regions. Interestingly, at the 0.1 Gy dose genes associated with upregulated 5hmC and 5mC showed the most overlap while this overlap was not significant at the 0.2 Gy dose. It is conceivable that this relationship reflects increased Tet-mediated demethylation at 0.2 Gy because regions with decreased 5mC and 5hmC had the most significant overlap at this dose.

### Gene expression in hippocampus following sham and ^56^Fe irradiation

**Fig. 7:**
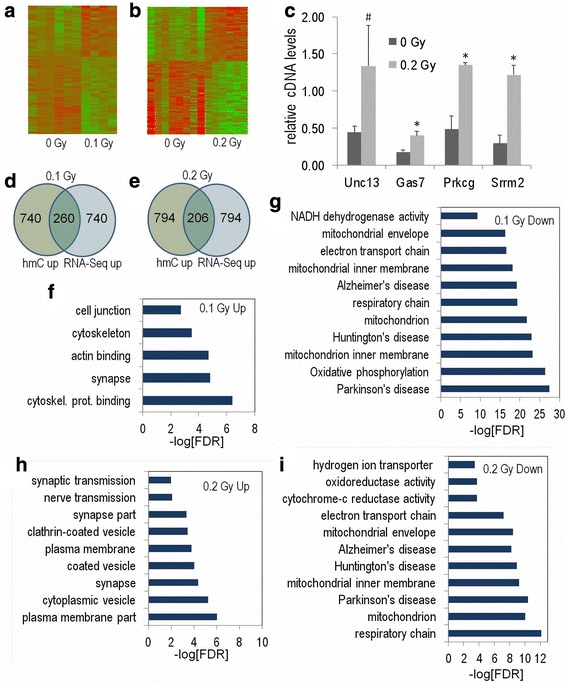
RNA-Seq analysis of the ^56^Fe ion-irradiated hippocampus at the 2-week time point. Heatmaps depict significantly-regulated genes (FDR-adjusted *p* < 0.05) at the 0.1 (**a**) and 0.2 (**b**) Gy dose. **c** Real-time PCR validation of significantly upregulated RNA-Seq regions at the 0.2 Gy 2wk time point (samples selected based on ranked *p*-value). Real-time PCR was conducted with absolute standard curves and 4 cDNA biological replicates. * denotes *p* < 0.05 and ^#^ denotes *p* < 0.1. **d** and **e** Overlap of top 1000 significantly-upregulated hmC regions and significantly-upregulated genes at the 0.1 (**d**) and 0.2 (**e**) Gy dose (within 50 kb of RefSeq TSS). At both doses, there was a highly significant overlap between regions with increased 5hmC and increased RNA transcription. **f**–**i** Gene ontology for the indicated significantly differentially-regulated regions (within 25 kb of RefSeq TSS). For the increased RNA transcription condition, there was more enrichment in pathways for the 0.2 (**f**) than the 0.1 (**h**) Gy dose. For the decreased RNA transcription condition, there was a highly significant enrichment for categories linked to neurodegenerative diseases, such as Alzheimer’s diseases, Parkinson’s disease, and Huntington’s disease, at the 0.1 (**g**) and 0.2 (**i**) Gy doses

**Fig. 8:**
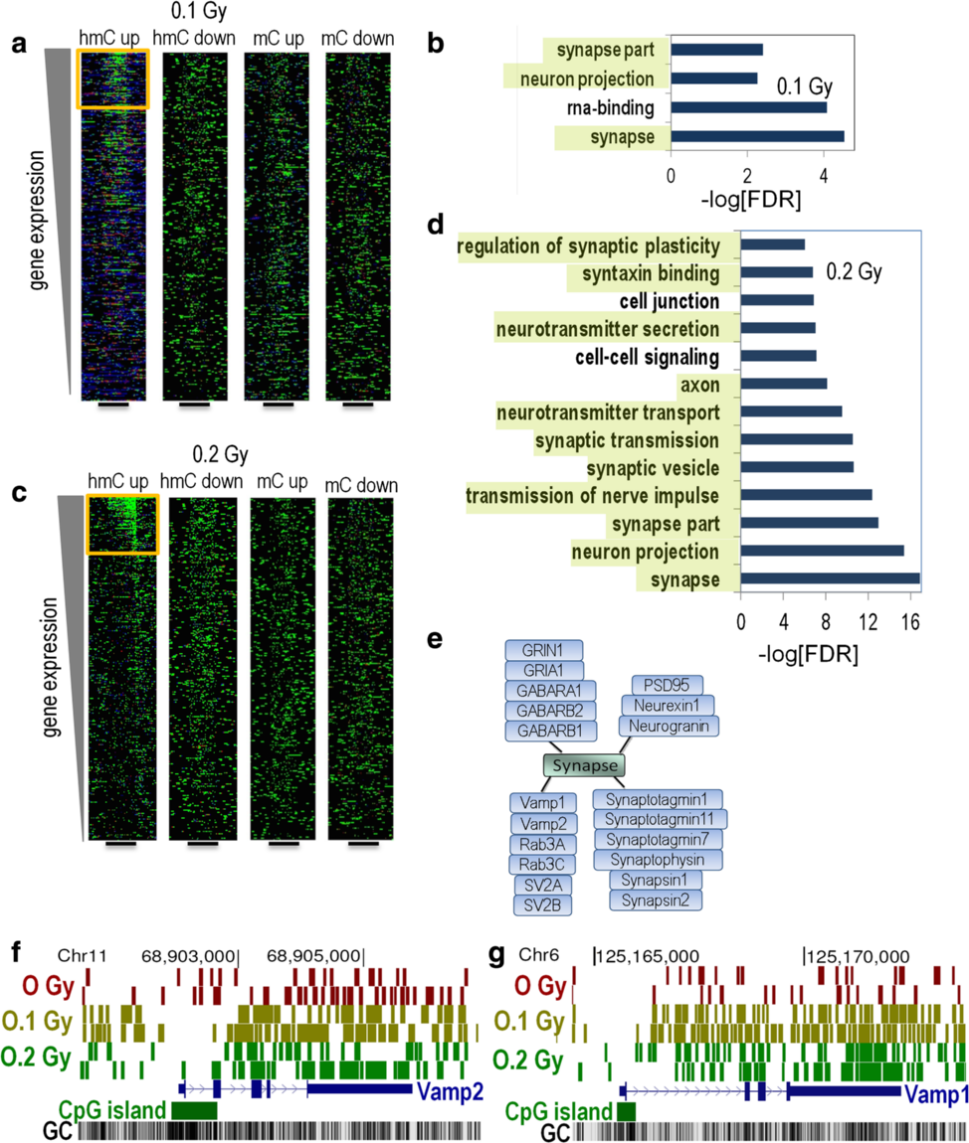
Correlation between alterations in DNA methylation and gene expression. **a** and **c** Heatmaps shows sequence density of significantly-regulated 5hmC regions sorted by RNA-Seq gene expression levels for the 0.1 (**a**) and 0.2 (**c**) Gy dose. Note the cluster of upregulated DIP-Seq data in intragenic regions at the top of the plot. The black bar denotes the position of scaled RefSeq intragenic regions. **b** and **d** Gene ontology analyses of the top 500 most up-regulated DIP-Seq regions (by normalized intragenic sequence difference) in the corresponding heatmaps of the 0.1 (**b**) and 0.2 (**d**) Gy doses. **e** Diagram depicts a subset of genes present in the synapse gene ontology category in D. Note the orthologs and preo/post-synaptic components. **f** and **g** UCSC genome browser track depicts DIP-Seq sequence density at two differentially hydroxymethylated gene selected from panels **b** and **d** Hatch marks may represent multiple sequences

Figure 7 illustrates heatmaps depicting significantly-regulated genes (FDR-adjusted *p* < 0.01) at 0.1 (a) and 0.2 Gy (b) at the two week time point. Real-time PCR validation of significantly upregulated genes at the 0.2 Gy dose confirmed that all genes showed the expected increase with 3 of 4 significantly increased (Fig. 7c). We examined the overlap between the top 1000 significantly regulated RNA-Seq genes and top 1000 annotated DIP-Seq regions. Interestingly, significant overlap at 0.1 and 0.2 Gy doses (*p* < 2x10^−16^ for both) was only seen for regions where both 5hmC signal and RNA transcription were positively correlated (Fig. 7d, e). For the increased RNA transcription condition, there was more enrichment in pathways for the 0.2 Gy (Fig. 7h) than the 0.1 Gy (Fig. 7f) dose. For the decreased RNA transcription condition, there was a highly significant enrichment for categories linked to neurodegenerative diseases such as Alzheimer’s disease, Parkinson’s disease, and Huntington’s disease at the 0.1 Gy (Fig. 7g; FDR-adjusted *p* < 1×10^−19^) and 0.2 Gy (Fig. 8h; FDR-adjusted *p* < 1×10^−10^) doses. Interestingly, the enrichment for neurodegenerative disease categories was markedly more significant at 0.1 Gy than 0.2 Gy (0.1 Gy: *p* < 1×10^−20^_;_ 0.2 Gy: *p* < 1×10^−9^). The RNA-Seq data for the 20-week time point did not show enrichment for pathways related to neurodegenerative disease (*p* > 0.01). Significantly-regulated Kegg pathway data for the decreased RNA transcription condition for the 0.1 Gy dose are illustrated for Alzheimer’s disease (Additional file 3: Figure S2), Parkinson’s disease (Additional file 4: Figure S3), and oxidative phosphorylation (Additional file 5: Figure S4).

To identify DNA methylation and gene expression correlations, we sorted the density of 5mC and 5hmC regions that showed significant changes by gene expression. Interestingly, regions that showed significant increases in 5hmC at 0.1 (Fig. 8a) and 0.2 (Fig. 8c) Gy clustered with highly expressed genes. The heatmap cluster of increased 5hmC showed remarkable spatial specificity and no similar clustering was seen for other 5hmC comparisons or for 5mC (Fig. 8a and c).

**Fig. 9:**
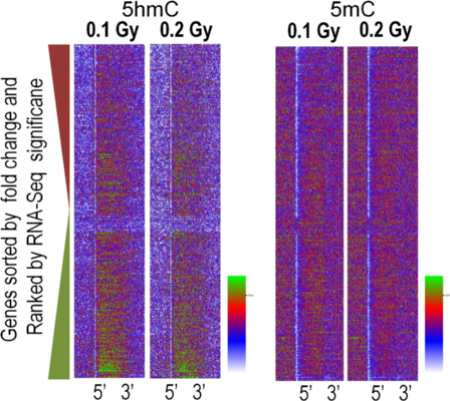
Heatmaps depict the density of 5hmC and 5mC signals sorted by significantly regulated RefSeq genes. Note the enrichment for increases in 5hmC in the gene-bodies of genes that were most significantly up-regulated by radiation. This was not seen for 5mC. 5’ and 3’ denote the boundaries of the scaled RefSeq annotation

To test whether the gene expression sorted 5hmC cluster might reflect biologically relevant pathways we performed gene ontology analyses. We selected the top 500 5hmC DIP-Seq regions from the heatmap cluster (by normalized intragenic sequence difference). The clustered 5hmC DMRs were significantly enriched for synapse-associated GO categories, with a very striking enrichment at the 0.2 Gy (Fig. 8d) as compared to 0.1 Gy (Fig. 8b) dose. This result suggests increased synapse remodeling/repair at the 0.2 Gy dose. The diagram in Fig. 8e illustrates a subset of genes in the synapse gene ontology category at the 0.2 Gy dose. UCSC genome browser track depicts 5hmC DIP-Seq data density at two differentially hydroxymethylated genes selected from the synapse gene ontology category in panels B an D (Fig. 8f and g). We further explored the positive relationship between 5hmC DMRs and gene expression by analyzing the density of 5hmC and 5mC signals sorted by significantly regulated RefSeq genes (Fig. 9). This analysis utilizes the converse relationship of our analysis above and it also detected specific enrichment for increases in 5hmC in the gene-bodies of genes that were most significantly up-regulated by ^56^Fe ion irradiation. This was not seen for 5mC (Fig. 9). Taken together, our results suggest that ^56^Fe ion irradiation triggers the coordinated upregulation of intragenic 5hmC and transcription for a subset of genes that play a key role in synapse function and development.

### Tet2 immunohistochemistry

**Fig. 10:**
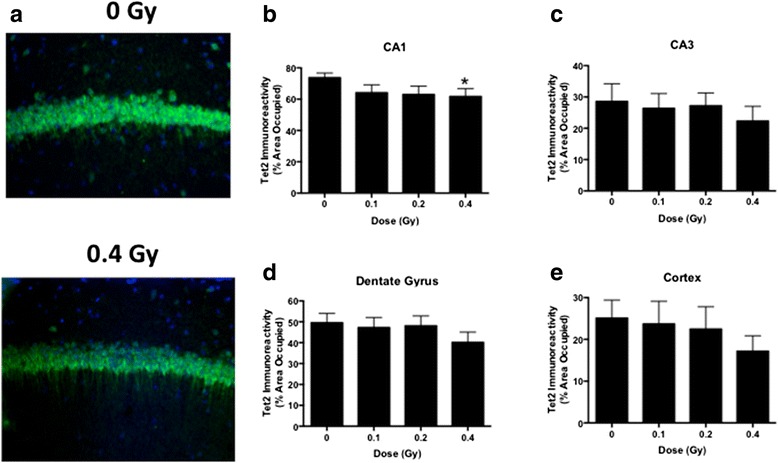
Tet2 immunoreactivity two weeks following sham or ^56^Fe-irradiation. **a** Representative images of Tet2 immunoreactivity in the CA1 region of the hippocampus of a sham-irradiated mouse and a mouse irradiated with 0.4 Gy. **b** Tet2 immunoreactivity levels in the CA1 region of the hippocampus. *p < 0.05 versus sham-irradiation. **c** Tet2 immunoreactivity levels in the CA3 region of the hippocampus. **d** Tet2 immunoreactivity levels in the dentate gyrus. **e** Tet2 immunoreactivity levels in the CA1 region of the cortex. *N* = 21 mice/dose. Scale bar: 100 μm

Finally, we analyzed immunoreactivity of TET2, one of the three TET enzymes responsible for converting 5mC to 5hmC [36] and likely the most important for brain function [37, 38, 51, 52], in the hippocampus and cortex two weeks following ^56^Fe ion irradiation. In the CA1 region of the hippocampus, Tet2 immunoreactivity was slightly but significantly lower following irradiation at 0.4 Gy than sham irradiation (Fig. 10a, b). In addition, there was a trend towards less Tet2 immunoreactivity in the CA1 of mice irradiated at 0.2 Gy than sham-irradiated mice (*p* = 0.0519, Fig. 10b). Although the general pattern of less Tet2 immunoreactivity in irradiated than sham-irradiated mice was the same in the CA3 region of the hippocampus (Fig. 10c), dentate gyrus (Fig. 10d), and cortex (Fig. 10e), it did not reach significance. There was also a trend towards less Tet2 immunoreactivity in the cortex of mice irradiated at 0.4 Gy than sham-irradiated mice (*p* = 0.0923, Fig. 10e). In contrast to the 2-week time point, there were no effects of ^56^Fe irradiation on Tet2 immunoreactivity at the 20-week time point.

## Discussion

In this study, we examined the short- and long-term effects of whole body ^56^Fe ion irradiation on hippocampus-dependent cognitive function, hippocampal DNA methylation, and hippocampal RNA expression. At the two-week time point, object recognition and network stability (defined by *Arc* mRNA localization) were impaired following irradiation at the 0.1 and 0.4 Gy doses, but not following irradiation at the 0.2 Gy dose. In contrast, no impairments in object recognition or network stability were seen at the 20-week time point for any of the tested doses. Consistent with this time-dependent pattern and suggesting substantial recovery, the significance of affected gene ontology for 5hmC was lower at the 20-week compared to the 2-week time point and no significant gene ontology pathways were observed for 5mC at the 20-week time point. Moreover, the 5hmC changes at the 2-week time point, but not the 5mC changes, tracked with gene expression changes detected with RNA-seq. Thus, while 5hmC changes were stable and/or represented new transcriptional activity, the changes in 5mC appeared transient and not linked to transcription changes. Because 5hmC is often found at promoters and transcribed regions of active genes, unlike 5mC, which is found at the promoters of inactive genes [53], these data support an important role for 5hmC in brain function following irradiation. The results from the 2-week time point suggest that the dynamic regulation of 5hmC and 5mC are coordinated, consistent with coupled DNA methylation-oxidation (Tet-mediated oxidation) and active demethylation.

Both 5mC and 5hmC in the brain are relatively persistent epigenetic marks [54, 55], but our recent work showed specific responses to radiation exposure for both DNA modifications [41]. That observation suggests an important role for DNA methylation (5mC and/or 5hmC) in brain function. The data presented here suggest further that 5hmC plays the more critical role for the long-term response to radiation exposure. Consistent with an important role for 5hmC in hippocampal function, we observed highly significant overlap for regions with increased 5hmC and increased RNA transcription at both the 0.1 and 0.2 Gy doses, with more enrichment in pathways for the 0.2 than the 0.1 Gy dose. This observation is consistent with the dose-dependent pattern in cognitive injury and suggests that the greater genomic and trancriptional response to the radiation damage at 0.2 Gy prevented the cognitive impairment observed at 0.1 Gy. For the decreased RNA transcription condition, there was a highly significant enrichment for categories linked to neurodegenerative diseases, such as Alzheimer’s disease, Parkinson’s disease (PD), and Huntington’s disease, and the decrease was greater at the 0.1 Gy dose; again consistent with increased cognitive impairment at this dose. This effect was transient, however, because there was no significant enrichment for categories linked to neurodegenerative disease for the 20-week time point nor were cognitive impairments observed. These data highlight the risk of transient radiation exposures leading to mission-critical cognitive impairments during space missions.

The gene ontology analyses of the top 500 most up-regulated DIP-Seq regions revealed that the clustered 5hmC DMRs were significantly enriched for synapse-associated GO categories, with a more profound enrichment at the 0.2 Gy than the 0.1 Gy dose. As noted above, this dose-dependent pattern suggests that cognitive performance in the mice was affected by the extent of the tissue response to the radiation injury. The mice irradiated with 0.1 Gy were cognitively impaired with regard to object recognition and network stability, while those irradiated with 0.2 Gy were not. Combined, these data suggest that damage caused at the 0.1 Gy dose was sufficient to cause cognitive injury, but not sufficient to induce synapse remodeling/repair that would have mitigated the cognitive injury. In contrast, we speculate that the higher level of damage at the 0.2 Gy dose induced a level of synapse remodeling/repair sufficient to mitigate the cognitive injury. According to this model, cognitive changes are observed when the synapse remodeling/repair response is insufficient to repair radiation damage. Consistent with this model, down-regulated RNA-Seq gene pathways linked to neuronal degeneration were more significantly enriched at 0.1 Gy than at the 0.2 Gy dose. Thus, decreases in these pathways may be associated with cognitive injury and impairment.

Unusual dose-dependent patterns, in which a lower dose causes more changes than a higher dose, are not restricted to ^56^Fe ion irradiation. When comparing the effects of ^28^Si ion irradiation (600 MeV), 0.25 Gy affected hippocampus-dependent contextual fear memory and this effect was associated with changes in synaptic plasticity not seen following irradiation at a dose of 1 Gy [2]. These unusual dose response patterns highlight the complexity of risk assessment regarding safe space radiation doses for astronauts. The fact that the 0.1 Gy dose of ^56^Fe ions is more detrimental for hippocampal function is especially concerning because this is a relevant dose for a deep space mission [56].

The pattern of the dose response for the effects of ^56^Fe ion irradiation on object recognition appears modulated by age at irradiation. In the current study, in which C57BL/6 J mice were irradiated with ^56^Fe ions at 6 months of age to better reflect the typical age of astronauts during space missions, those irradiated with ^56^Fe ions at 0.1 Gy were impaired in object recognition, while those irradiated at 0.2 Gy were not. In contrast to these data, when C57BL/6 J mice were irradiated at 2 months of age, both dose groups were impaired in object recognition [22]. These data indicate that for some cognitive measures and dose exposures, older adult mice might be less sensitive to effects of ^56^Fe ion irradiation than younger adult mice. Future efforts are warranted to assess why there is this age-dependency in sensitivity to develop cognitive injury following space irradiation.

The short- and long-term effects of ^56^Fe ion irradiation on DNA methylation could not be accounted for by radiation-induced changes in TET2 protein levels. The TET2 protein levels were only reduced significantly in the CA1 region of the hippocampus following exposure to the 0.4 Gy dose. Comparing TET2 immunoreactivity across brain regions, the levels were particularly high in the CA1 region of the hippocampus and high in the dentate gyrus, with much lower levels in the CA3 region of the hippocampus and cortex. The high levels of TET2 in the CA1 region of the hippocampus is interesting, as the effects of ^56^Fe ion irradiation on network stability likely related to hippocampal place cells was also seen in this hippocampal area. The high levels of TET2 in the hippocampus are consistent with other studies showing that brain TET 2 protein levels are higher than those of TET1 or TET3 [37, 38].

We did not see alterations in DNA methylation or RNA transcription of the *Arc* gene following ^56^Fe ion irradiation, suggesting that *Arc* is not a direct target of epigenetic regulation under these exposure conditions. This is not surprising because the observed changes in *Arc* expression in the CA1 region of the hippocampus dealt with nuclear vs. cytoplasmic RNA localization. The consistency between the impairments in network stability, as assessed by *Arc* mRNA localization data in the CA1 region of the hippocampus and impairments in novel object recognition at 0.1 but not 0.2 Gy, and the differences in DNA methylation at these two doses is striking and underlines the value of dose dependency of hippocampal impairments following ^56^Fe ion irradiation.

Some of the alterations in DNA methylation at the 2- and 20-week time point were not specific for ^56^Fe ion irradiation and also seen at the 20-week time point following proton irradiation in another study [41]. For example, the non-neuronal category of cell adhesion was seen as a significant pathway for 0.1 Gy 5hmC up and 5hmC down at the 2- and 20-week time points and for 5mC down at the 2-week time point. These space irradiation-induced long-term changes in DNA methylation might interact with age-dependent changes in DNA methylation that are thought to be associated with the risk to develop various neurological conditions [57–60] and thereby increase the susceptibility of the brain to detrimental effects of additional radiation exposure during long duration missions.

The distinct hippocampal outcome measures used in this study showed differential susceptibility to detrimental effects of ^56^Fe ion irradiation. In contrast to impairments in object recognition and network stability, no cognitive changes were seen in spatial learning and memory in the water maze or in habituation to the open field. There was a 24-h interval between training and assessment of memory retention in the object recognition test, while in the water maze training and assessment of memory retention occurred on the same day. These data suggest that a longer time interval and potentially protein synthesis, which is required for long-term memory [61], might be required to detect detrimental effects of ^56^Fe ion irradiation on memory retention in 6-month-old mice. However, spatial habituation learning also involved 24-h intervals and did not show profound radiation effects, while the assessments of network stability which involved two tests separated by only 25 min did reveal detrimental effects of ^56^Fe ion irradiation. In total, our cognitive results highlight the importance of considering multiple hippocampal outcome measures in assessing effects of space irradiation on hippocampal function.

## Conclusions

In summary, ^56^Fe ion irradiation at a dose as low as 0.1 Gy impairs object recognition and network stability, and is associated with short- and long-term alterations in 5hmC and short-term changes in 5mC in the hippocampus. Object recognition and network stability were not significantly affected at 0.2 Gy, a dose at which 5hmC DMRs were significantly enriched for synapse-associated gene ontology categories. Taken together, the 0.1 and 0.2 Gy data suggest that synapse remodeling coupled with epigenomic remodeling explains how the hippocampus responds to radiation exposure, and that a potentially dangerous window of exposure exists that is sufficient to induce cognitive change without triggering a sufficient transcriptional response that would prevent this change. Our data also suggest that 5hmC remodeling is both more stable than 5mC remodeling and is highly correlated with significant changes in gene expression. Thus, our study suggests that 5hmC is a sensitive biomarker of beneficial and adverse outcome pathways. Future efforts are warranted to determine the effects of different kinds of irradiation astronauts will encounter in the space environment on hippocampus-dependent cognitive function and hippocampal DNA methylation, how these changes interact, and finally how they occur.

## Additional files

Additional file 1: Figure S1.
**A.** Open field activity at the 2-week time point. There was an effect of day (*p* < 0.001). While all groups habituated to the open field and showed higher activity levels in the open field on the first day than subsequent days, the mice irradiated with ^56^Fe ions (600 MeV) at 0.1 Gy moved less than sham-irradiated mice (*p* = 0.044). **B.** Open field activity at the 20-week time point. There was an effect of day (*p* < 0.001), with higher activity levels on the first day than subsequent days. On day 2, activity levels were higher in mice irradiated with ^56^Fe ions (600 MeV) at 0.2 Gy (*p* = 0.024) and that on day 3, mice irradiated with ^56^Fe ions (600 MeV) at 0.1 Gy (*p* = 0.044) or 0.4 Gy (*p* = 0.046) were higher than those in sham-irradiated mice. *N* = 16 mice/dose. **p* < 0.05 versus sham-irradiation. (TIFF 728 kb)
Additional file 2: Table S1. The percentage of Arc-positive neurons in the dentate gyrus at the 2-week time point^1^. (DOCX 96 kb)
Additional file 3: Figure S2.
**A.** Significantly regulated Kegg pathway data for the decreased RNA transcription condition for the 0.1 Gy dose are illustrated for Alzheimer’s disease (AD). Key molecules in AD identified included Amyloid Precursor Protein (APP), β-Secretase (BACE), presenilin (PSEN), insulin-degrading enzyme (IDE), and apolipoprotein E (apoE). (TIFF 10447 kb)
Additional file 4: Figure S3. Significantly regulated Kegg pathway data for the decreased RNA transcription condition for the 0.1 Gy dose are illustrated for Parkinson’s disease (PD). Key molecules in PD identified included the dopamine transporter (DAT), Parkin, tyrosine hydroxylase (TH), and molecules playing a role in mitochondrial pathways. (TIFF 13247 kb)
Additional file 5: Figure S4. Significantly regulated Kegg pathway data for the decreased RNA transcription condition for the 0.1 Gy dose are illustrated for oxidative phosphorylation. Key molecules identified included NADH dehydrogenase, Cytochrome c oxidase, and F-type ATPase. (TIFF 9302 kb)
