# Supplementary figures and images for: Short- and long-term effects of 56Fe irradiation on cognition and hippocampal DNA methylation and gene expression
Source: BMC Genomics. 2016 Oct 24;17:825. doi: 10.1186/s12864-016-3110-7 (PMC5078898; doi:10.1186/s12864-016-3110-7)

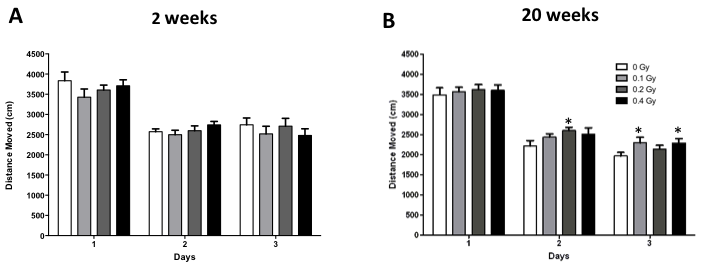

Supplement: Additional file 1: Figure S1. — A. Open field activity at the 2-week time point. There was an effect of day (p < 0.001). While all groups habituated to the open field and showed higher activity levels in the open field on the first day than subsequent days, the mice irradiated with 56Fe ions (600 MeV) at 0.1 Gy moved less than sham-irradiated mice (p = 0.044). B. Open field activity at the 20-week time point. There was an effect of day (p < 0.001), with higher activity levels on the first day than subsequent days. On day 2, activity levels were higher in mice irradiated with 56Fe ions (600 MeV) at 0.2 Gy (p = 0.024) and that on day 3, mice irradiated with 56Fe ions (600 MeV) at 0.1 Gy (p = 0.044) or 0.4 Gy (p = 0.046) were higher than those in sham-irradiated mice. N = 16 mice/dose. *p < 0.05 versus sham-irradiation. (TIFF 728 kb) [file 12864_2016_3110_MOESM1_ESM.tiff]

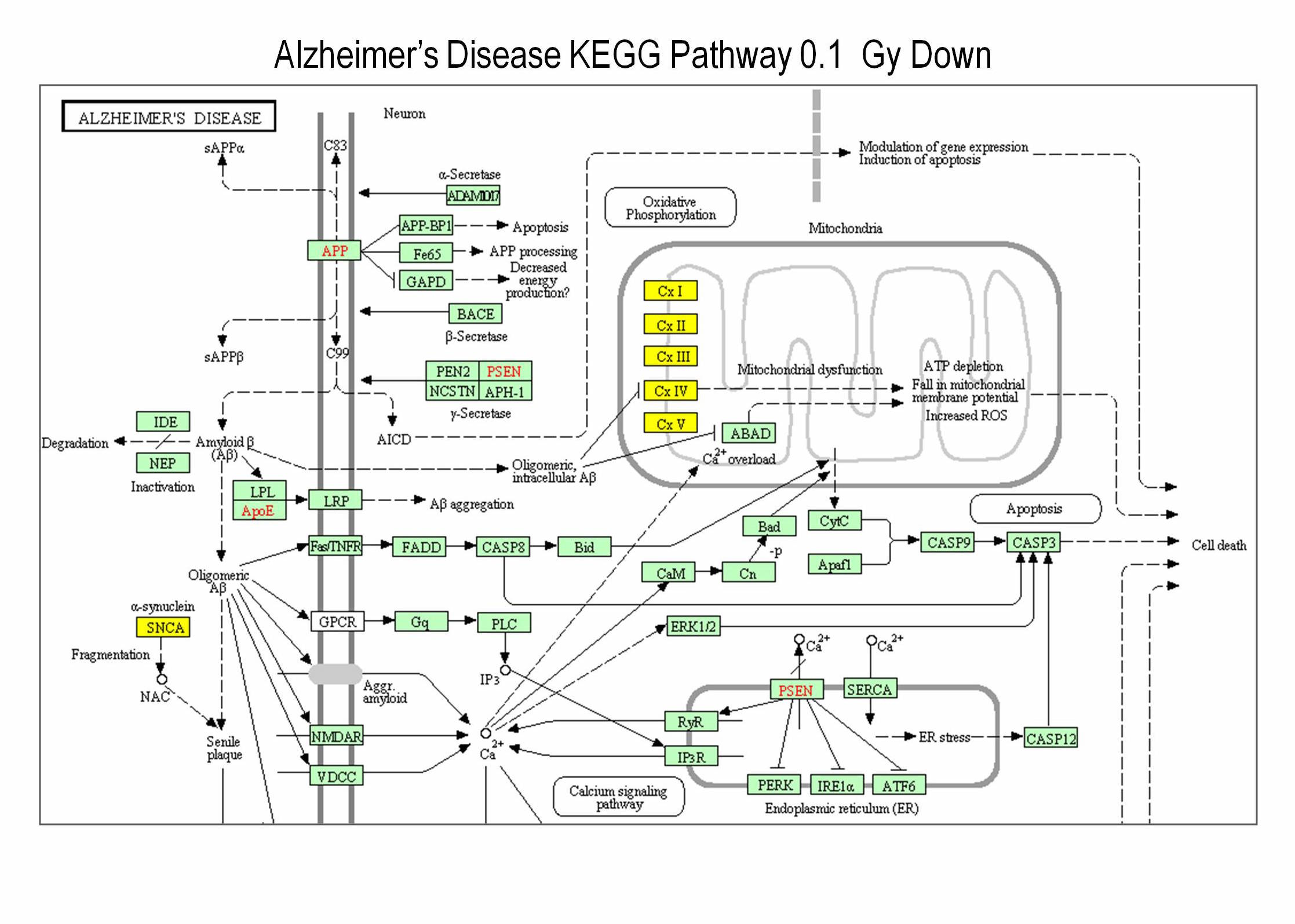

Supplement: Additional file 3: Figure S2. — A. Significantly regulated Kegg pathway data for the decreased RNA transcription condition for the 0.1 Gy dose are illustrated for Alzheimer’s disease (AD). Key molecules in AD identified included Amyloid Precursor Protein (APP), β-Secretase (BACE), presenilin (PSEN), insulin-degrading enzyme (IDE), and apolipoprotein E (apoE). (TIFF 10447 kb) [file 12864_2016_3110_MOESM3_ESM.tiff]

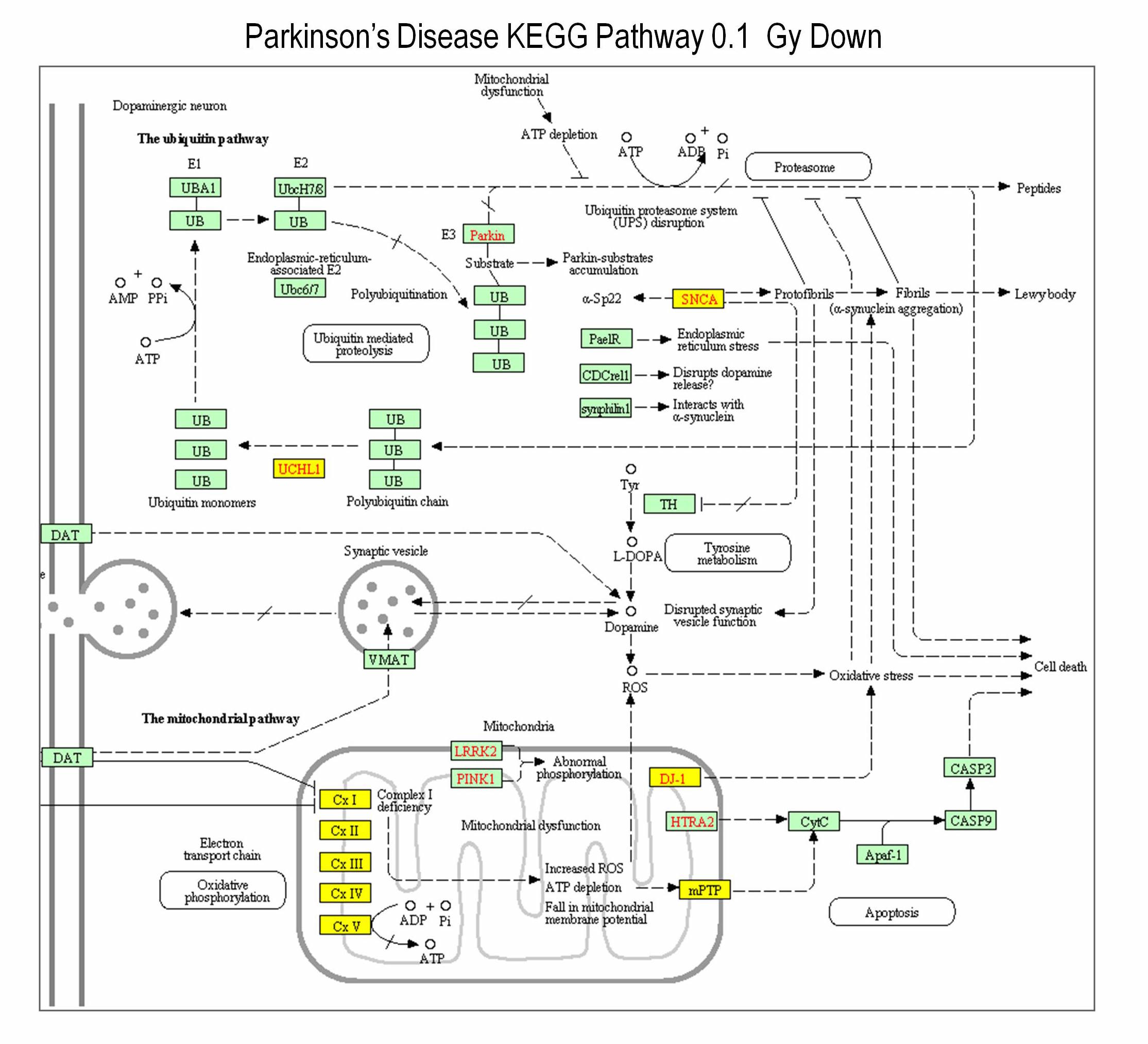

Supplement: Additional file 4: Figure S3. — Significantly regulated Kegg pathway data for the decreased RNA transcription condition for the 0.1 Gy dose are illustrated for Parkinson’s disease (PD). Key molecules in PD identified included the dopamine transporter (DAT), Parkin, tyrosine hydroxylase (TH), and molecules playing a role in mitochondrial pathways. (TIFF 13247 kb) [file 12864_2016_3110_MOESM4_ESM.tiff]

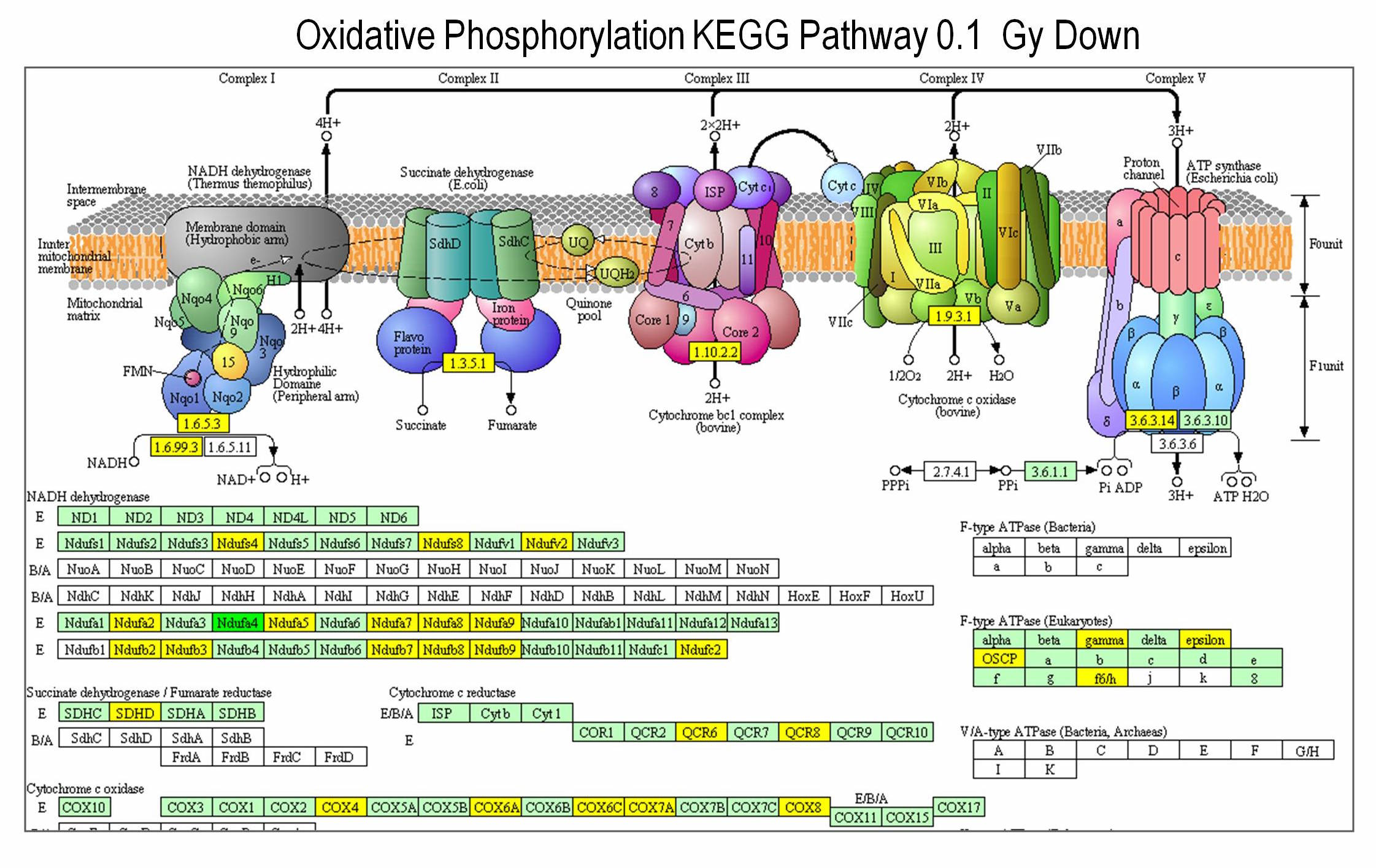

Supplement: Additional file 5: Figure S4. — Significantly regulated Kegg pathway data for the decreased RNA transcription condition for the 0.1 Gy dose are illustrated for oxidative phosphorylation. Key molecules identified included NADH dehydrogenase, Cytochrome c oxidase, and F-type ATPase. (TIFF 9302 kb) [file 12864_2016_3110_MOESM5_ESM.tiff]
