# Supplementary material for: Short- and long-term effects of 56Fe irradiation on cognition and hippocampal DNA methylation and gene expression
Source: BMC Genomics. 2016 Oct 24;17:825. doi: 10.1186/s12864-016-3110-7 (PMC5078898; doi:10.1186/s12864-016-3110-7)
Supplement: Additional file 2: Table S1. — The percentage of Arc-positive neurons in the dentate gyrus at the 2-week time point1. (DOCX 96 kb) [file 12864_2016_3110_MOESM2_ESM.docx]

*Supplementary Figure Legends*

**Suppl. Fig. 1. A.** Open field activity at the 2-week time point. There was an effect of day (*p* < 0.001). While all groups habituated to the open field and showed higher activity levels in the open field on the first day than subsequent days, the mice irradiated with ^56^Fe ions (600 MeV) at 0.1 Gy moved less than sham-irradiated mice (*p* = 0.044). **B.** Open field activity at the 20-week time point. There was an effect of day (*p* < 0.001), with higher activity levels on the first day than subsequent days. On day 2, activity levels were higher in mice irradiated with ^56^Fe ions (600 MeV) at 0.2 Gy (*p* = 0.024) and that on day 3, mice irradiated with ^56^Fe ions (600 MeV) at 0.1 Gy (*p* = 0.044) or 0.4 Gy (*p* = 0.046) were higher than those in sham-irradiated mice. *N* = 16 mice/dose. **p* < 0.05 versus sham-irradiation.

Suppl. Fig. 2A. Significantly regulated Kegg pathway data for the decreased RNA transcription condition for the 0.1 Gy dose are illustrated for Alzheimer’s disease (AD). Key molecules in AD identified included Amyloid Precursor Protein (APP), β-Secretase (BACE), presenilin (PSEN), insulin-degrading enzyme (IDE), and apolipoprotein E (apoE).

Suppl. Fig. 3. Significantly regulated Kegg pathway data for the decreased RNA transcription condition for the 0.1 Gy dose are illustrated for Parkinson’s disease (PD). Key molecules in PD identified included the dopamine transporter (DAT), Parkin, tyrosine hydroxylase (TH), and molecules playing a role in mitochondrial pathways.

Suppl. Fig. 4. Significantly regulated Kegg pathway data for the decreased RNA transcription condition for the 0.1 Gy dose are illustrated for oxidative phosphorylation. Key molecules identified included NADH dehydrogenase, Cytochrome c oxidase, and F-type ATPase.

**Supplementary Table 1.** The percentage of Arc-positive neurons in the dentate gyrus at the 2-week time point^1^.

| Dose (Gy) | Enclosed Blade; same environment | Enclosed Blade; different environments | Free Blade; same environment | Free Blade; different environments |
| --- | --- | --- | --- | --- |
| 0 | 1.54 ± 0.17 | 1.79 ± 0.21 | 1.25 ± 0.18 | 1.53 ± 0.20 |
| 0.1 | 1.57 ± 0.09 | 1.36 ± 0.21 | 1.36 ± 0.15 | 0.93 ± 0.23 |
| 0.2 | 1.71 ± 0.30 | 1.54 ± 0.25 | 0.99 ± 0.24 | 1.12 ± 0.27 |
| 0.4 | 1.64 ± 0.15 | 1.46 ± 0.23 | 1.44 ± 0.16 | 1.32 ± 0.25 |

^1^ *N* = 5-8 mice/dose/environment.
